# Epigenetic alterations in cytochrome P450 oxidoreductase (*Por*) in sperm of rats exposed to tetrahydrocannabinol (THC)

**DOI:** 10.1038/s41598-020-69204-7

**Published:** 2020-07-23

**Authors:** Kelly S. Acharya, Rose Schrott, Carole Grenier, Zhiqing Huang, Zade Holloway, Andrew Hawkey, Edward D. Levin, Susan K. Murphy

**Affiliations:** 1 Department of Obstetrics and Gynecology, Duke University Medical Center, 701 West Main Street, Suite 510, Durham, NC 27701 USA; 2 Duke Nicholas School of the Environment, University Program in Environmental Health, Durham, NC USA; 3 Department of Psychiatry and Behavioral Sciences, Duke University Medical Center, Durham, NC USA

**Keywords:** Epigenetic memory, Translational research

## Abstract

As marijuana legalization is increasing, research regarding possible long-term risks for users and their offspring is needed. Little data exists on effects of paternal tetrahydrocannabinol (THC) exposure prior to reproduction. This study determined if chronic THC exposure alters sperm DNA methylation (DNAm) and if such effects are intergenerationally transmitted. Adult male rats underwent oral gavage with THC or vehicle control. Differentially methylated (DM) loci in motile sperm were identified using reduced representation bisulfite sequencing (RRBS). Another cohort was injected with vehicle or THC, and sperm DNAm was analyzed. Finally, THC-exposed and control adult male rats were mated with THC-naïve females. DNAm levels of target genes in brain tissues of the offspring were determined by pyrosequencing. RRBS identified 2,940 DM CpGs mapping to 627 genes. Significant hypermethylation was confirmed (*p* < 0.05) following oral THC administration for cytochrome P450 oxidoreductase (*Por*), involved in toxin processing and disorders of sexual development. *Por* hypermethylation was not observed after THC injection or in the subsequent generation. These results support that THC alters DNAm in sperm and that route of exposure can have differential effects. Although we did not observe evidence of intergenerational transmission of the DNAm change, larger studies are required to definitively exclude this possibility.

## Introduction

In the United States, legalization of marijuana (*Cannabis sativa*) is increasing; as of August 2019, 33 states have legalized medical marijuana, with 11 of those also legalizing recreational marijuana^1^. Meanwhile, the perception of the safety of cannabis use has also been increasing, with more adolescents and adults stating the perceived risk associated with marijuana to be low to none^2^, and almost three quarters of people of ages 21–37 supporting legalization^3^. Existing studies regarding the safety of marijuana in individuals of reproductive age typically examine effects of use during puberty/childhood on adult outcomes as well as maternal marijuana use during pregnancy. There are multiple studies on the use of marijuana and its association with fertility, including studies that found lower sperm concentrations in men who regularly use marijuana^4,5^. However, the effect on actual fecundity and time to conception has been questioned^6^. There is a paucity of data surrounding preconception use of marijuana by the mother or father and the effects of this preconception use on future generations of offspring.

### Inter- and transgenerational effects of THC exposure

When studying male preconception environmental exposures, “intergenerational” inheritance studies examine the effects on the progeny of the exposed (F0) males; in other words, the term intergenerational refers to the subsequent (F1) generation (which was exposed to the environmental influence as a gamete). “Transgenerational” inheritance refers to the subsequent (F2) generation, or individuals who were never exposed themselves including as gametes. At least two existing rodent studies have shown an intergenerational effect of THC exposure. In a paper by Szutorisz et al. in 2014, adolescent male and female rats were exposed to THC; these rats were then bred in adulthood, and their adult offspring were found to have altered striatal synaptic plasticity and increased work effort to self-administer heroin when compared with offspring of unexposed rats^7^. The same group later found that offspring of rats exposed to THC had altered DNA methylation profiles in the nucleus accumbens, a region of the brain known to function in reward processing and addiction^8^. However, each of those studies exposed both male and female rats to THC and did not differentiate between maternally- versus paternally-inherited epigenetic alterations. Most recently, our group found that genome-scale methylation patterns are altered in the sperm of rats (and humans) exposed to THC, supporting the idea that through an altered sperm epigenome, THC-related effects may be passed to future generations^5^.

The idea of intergenerational or transgenerational inheritance of methylation changes due to environmental exposures is confounded by the natural process of methylation reprogramming, which occurs in humans and rodents. In human embryos at around post-fertilization week 4, the primordial germ cells migrate to the developing genital ridges and undergo “reprogramming,” or genome-wide demethylation, to erase epigenetic marks that were present in the prior generation. Shortly after erasure, most of the DNA methylation marks are re-established but then are finalized in males during the process of spermatogenesis, which commences at adolescence. Sperm DNA methylation continues to be remodeled throughout spermatogenesis (including during a final period of epididymal maturation) during an individual’s reproductive years, rendering the sperm epigenome susceptible to environmental exposures during all stages of spermatogenesis in adults. Another epigenetic reprogramming event occurs immediately following fertilization, whereby the paternal pronucleus is actively demethylated and the maternal pronucleus demethylates more passively over the course of early cell divisions. In this case, imprinted genes and repetitive regions retain the methylation patterns established in the primordial germ cells^9^. Under the presumption that erasure is genome-wide, we expect that any epigenetic alterations incurred by an individual after an environmental exposure, for example, would be erased during post-fertilization reprogramming and could not be passed to the next generation. However, a relatively large number of non-imprinted, non-repetitive loci have been found to escape this process and retain their epigenetic marks during reprogramming, so-called methylation “escapees”^10^. Presumably, if a gene falls within this category and the individual’s gametes incur altered methylation through environmental exposure, the methylation changes may be passed on to future generations through inter- or transgenerational inheritance, providing a biologically-plausible mechanism by which environmental exposures of one generation may cause phenotypic changes in subsequent generation(s).

### Route of exposure

Δ^9^-tetrahydrocannabinol (THC) is the primary psychoactive component of *Cannabis sativa*. Considerable efforts have focused on determining the pharmacokinetic and pharmacodynamic properties specific to the route of administration of THC, especially in regard to synthetic THC used for medical purposes, with the aim to maximize medicinal properties while minimizing toxicity and unwanted neurologic side effects such as anxiety and acute paranoia. Inhalation is the primary and preferred route of administration for cannabis users, as this rapidly delivers THC from the lungs to the brain, and THC reaches higher peak levels more quickly than with oral administration of THC, which contributes to its abuse potential. The inhaled THC is absorbed into the blood, is metabolized in the liver and is later excreted as metabolites in feces (80%) and urine (20%). Oral formulations of THC (for example, synthetic THC marketed as dronabinol or *Marinol*®) are ingested and then undergo an extensive first-pass metabolism in the liver, with a slower distribution of drug in the tissues and a later peak plasma concentration as compared to inhalation. First-pass effect metabolizes THC into its acid metabolites, 11-nor-9-carboxytetrahydrocannabinol (THC-COOH) and 11-hydroxytetrahydrocannabinol (11-OH-THC), before the THC reaches its sites of action. The cytochrome P450 class of enzymes is active in metabolizing THC to THC-COOH and 11-OH-THC in the liver. This delayed onset of action, lower peak plasma levels, longer duration of effects and delayed return to baseline, all in part due to the lower oral bioavailability and the more extensive first-pass effect after ingestion, are significant ways in which oral administration of THC differs from the inhaled route. Studies have shown that after IV administration of radionucleotide-labeled THC to rats, higher THC levels were found in the lung than in other tissues, indicating that THC injections may be the best proxy for inhaled THC in animal models^11^. Furthermore, because of the difficulty of exact dosing of THC to rats housed with multiple animals per cage, oral gavage, rather than edibles, has been used to represent ingestion. Interestingly, after repeated and chronic exposures to THC, the chemical has been found to cross the blood-testis barrier^12^.

Given the current trend of cannabis legalization and the limited data regarding safety of preconception cannabis use in regards to reproductive outcomes and offspring health, the objective of this study was to investigate the effect of various routes of cannabis use on the epigenetic profile of sperm in male rats and to determine whether these epigenetic marks are intergenerationally inherited.

## Results

Reduced representation bisulfite sequencing (RRBS) revealed 2,940 CpG sites which were differentially methylated between sperm from THC exposed and unexposed rats. These sites mapped to 627 discrete genes; of these, five genes were identified for which at least five CpG sites were significantly differentially methylated between exposed rats and controls (Table 1). All of these CpG sites in these genes were hypermethylated in the sperm of the rats that had undergone oral gavage with THC. The five genes*, Adora2a, Cbx6, Hipk4, Mag*, and *Por,* were searched in Pubmed (www.pubmed.com), Genecards (www.genecards.org), and Genetics Home Reference (www.ghr.nlm.nih.gov) to ascertain their biologic function and relevance to either sperm function or early development. The gene with the highest number of differentially methylated CpG sites was *Por*, with nine CpG sites identified by RRBS. Two additional sites in the region were also captured in the RRBS data and found to be hypermethylated with THC exposure but did not differ significantly between exposed and unexposed sperm.

**Table 1:** Differentially methylated genes by RRBS selected for validation by bisulfite pyrosequencing.

Gene symbol	Gene name	# Significant CpG sites	Genomic coordinates	Effect of THC exposure	Gene product/function
*Adora2a*	Adenosine A2a receptor	7	*Chr 20	Hyper-methylation	Hippocampal volume, memory, caffeine response, anxiety, sexual dimorphism
			14,273,825		
			14,273,837		
			14,273,844		
			14,273,856		
			14,273,862		
			14,273,916		
			14,273,931		
*Cbx6*	Chromobox 6	5	Chr 7	Hyper-methylation	“Epigenetic regulator” in embryonic stem cells
			121,052,425		
			121,053,055		
			121,053,069		
			121,053,087		
			121,053,095		
*Hipk*	Homeodomain interacting protein kinase 4	5	Chr 1	Hyper-methylation	Modulator of cell stress response; posttranslational modifications (eg of p53)
			84,336,647		
			84,336,652		
			84,336,711		
			84,336,719		
			84,336,724		
*Mag*	Myelin associated glycoprotein	5	Chr 1	Hyper-methylation	Myelinated neuron cell–cell interactions; neuropathies
			89,348,074		
			89,348,079		
			89,348,127		
			89,348,149		
			89,348,159		
*Por*	Cytochrome P450 oxidoreductase	9	Chr 12	Hyper-methylation	Critical role in steroidogenesis, including formation of testosterone, estrogen, and glucocorticoids (cortisol). Essential for normal sexual development and reproduction. Electron donor in Krebs cycle, also functions in toxin metabolism. POR deficiency cause disorders of sexual development and infertility
			23,999,254		
			23,999,265		
			23,999,279		
			23,999,314		
			23,999,317		
			23,999,321		
			23,999,340		
			23,999,432		
			23,999,460		

*Chromosome.

**Figure 1:**
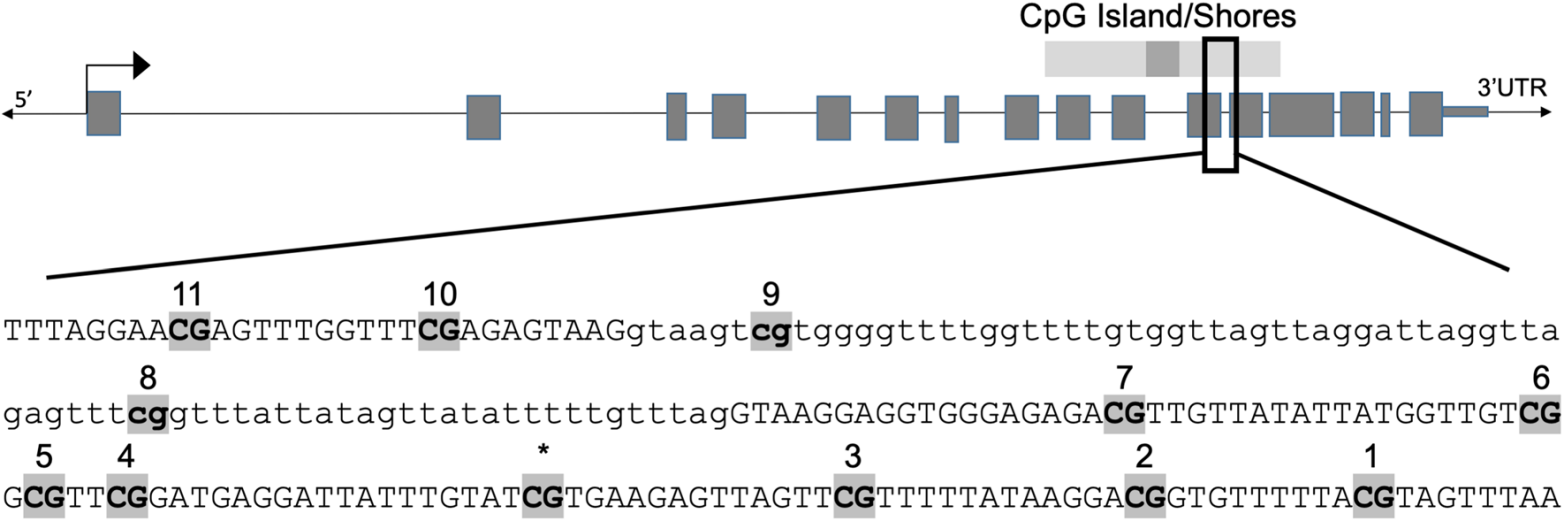
Gene structure and region of interest of rat *Por*, with exons represented by gray boxes, and introns represented by open line. The positions of the CpG island, in dark gray, and the CpG shores, in light gray, are shown above the gene. The sequence of the region of interest is shown, with exons represented by capital letters and the intron by lower case letters. The CpG sites of interest (as discovered by RRBS) are highlighted and in bold. The numbers above each site represent the CpG site number designation given in our targeted sequencing. The star represents one site which was included in the RRBS data but not included in any of the three pyrosequencing assays used to measure methylation at this region. *Por* assay 1 encompassed sites 11, 10, and 9. *Por* assay 2 covered sites 7, 6, 5, and 4. *Por* assay 3 included a new site not identified in RRBS (*) as well as sites 3, 2, and 1. Site 8 was not included in the targeted sequencing assays.

Bisulfite pyrosequencing assays were designed for *Adora2a*, *Cbx6*, *Hipk4* and *Mag,* and these assay designs were validated using defined mixtures of sodium bisulfite modified unmethylated and methylated rat genomic DNA to comprise 0, 25, 50, 75, and 100% methylation. The expected versus observed methylation was graphed for each assay, with all correlation coefficients > 0.96 (Supplemental Figure S1). These results indicate that pyrosequencing was able to discriminate increasing levels of methylation across the full range of possible values for all four genes. Because the nine CpG sites of interest in *Por* span 209 base pairs (bp), we designed and validated three pyrosequencing assays for this gene that were able to capture eight of these nine sites as well as three additional sites (Supplemental Figure S1), all with correlation coefficients > 0.96. The targeted sequencing assays captured one CpG site that was not included in the RRBS results, while the targeted sequencing did not encompass one CpG site which had been identified as significant in the RRBS results (Fig. 1).

**Figure 2:**
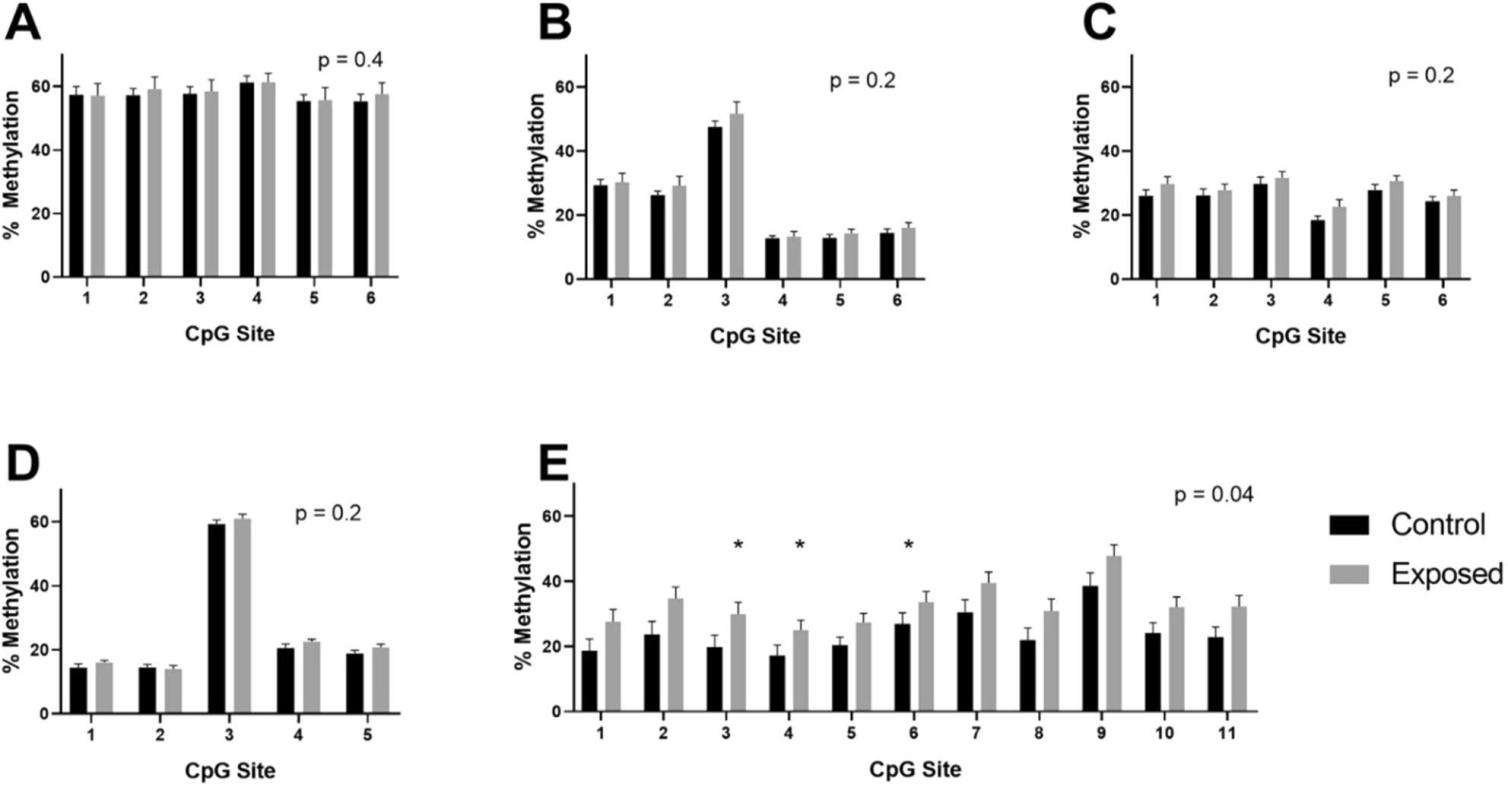
Pyrosequencing results for F0 control (black, n = 9) versus THC-exposed (gray, n = 7) sperm in *Adora2a* (**A**), *Cbx6* (**B**), *Hipk4* (**C**), *Mag* (**D**), and *Por* (**E**). Mean and standard error of the mean (SEM) are graphed. The x axis shows results for the CpG sites of interest in each gene, while the y axis shows methylation measured. Overall p values represent the results from one-tailed student’s t test of the mean of the methylation across all CpG sites measured in the exposed versus unexposed groups. *One-tailed t test *p* < 0.05.

The pyrosequencing assays showed that the directionality of methylation changes measured were consistent with the RRBS results, showing higher methylation in the THC-exposed sperm in all CpG sites analyzed with the exception of CpG site 1 for *Adora2a,* which showed increased average methylation in the control group, albeit not significant (Fig. 2). Four of the five genes (*Adora2a, Cbx6, Hipk4*, and *Mag*) showed a greatly decreased magnitude of effect size by targeted sequencing than what was detected by RRBS (Fig. 2A–D) (one-tailed *p* > 0.05). In contrast, *Por* showed hypermethylation at each CpG site, with methylation differences of 6.7–11.1% relative to controls (Fig. 2E). While the RRBS data showed a mean methylation difference across these CpG sites of 16.8% (41% in the THC exposed vs. 24.2% in the control sperm), the mean methylation as determined by pyrosequencing in the region of interest in *Por* in THC-exposed sperm was 32.8% versus 24.1% in control sperm, for a mean methylation difference of 8.7% (*p* = 0.04).

**Figure 3:**
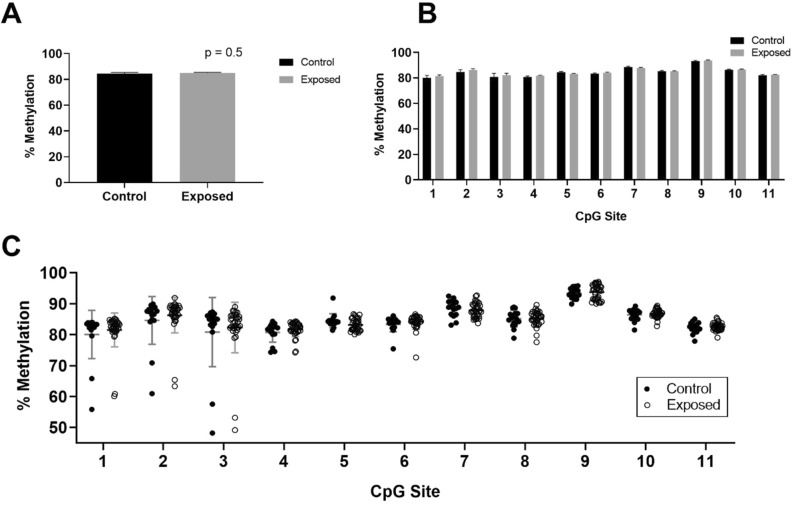
Intergenerational inheritance analysis. Methylation in the cerebral cortex of the F1 generation produced from control (black, n = 16) versus THC-exposed (gray, n = 35) F0 male rats. (**A**) Mean methylation (+ SEM) of the region of interest in *Por* of the offspring from parentally-unexposed versus exposed rats, (**B**) Methylation results (+ SEM) from (**A**) for the same population, displayed by individual CpG site. (**C**) Same data as in (**A**), except plotted to show methylation percentage at each CpG site in individual rats, with open circles representing progeny of THC-exposed fathers, while black dots represent the progeny of control fathers.

When the methylation of Por was examined in the brains of the F1 generation, we found no differences between THC exposed and control rats (Fig. 3).

**Figure 4:**
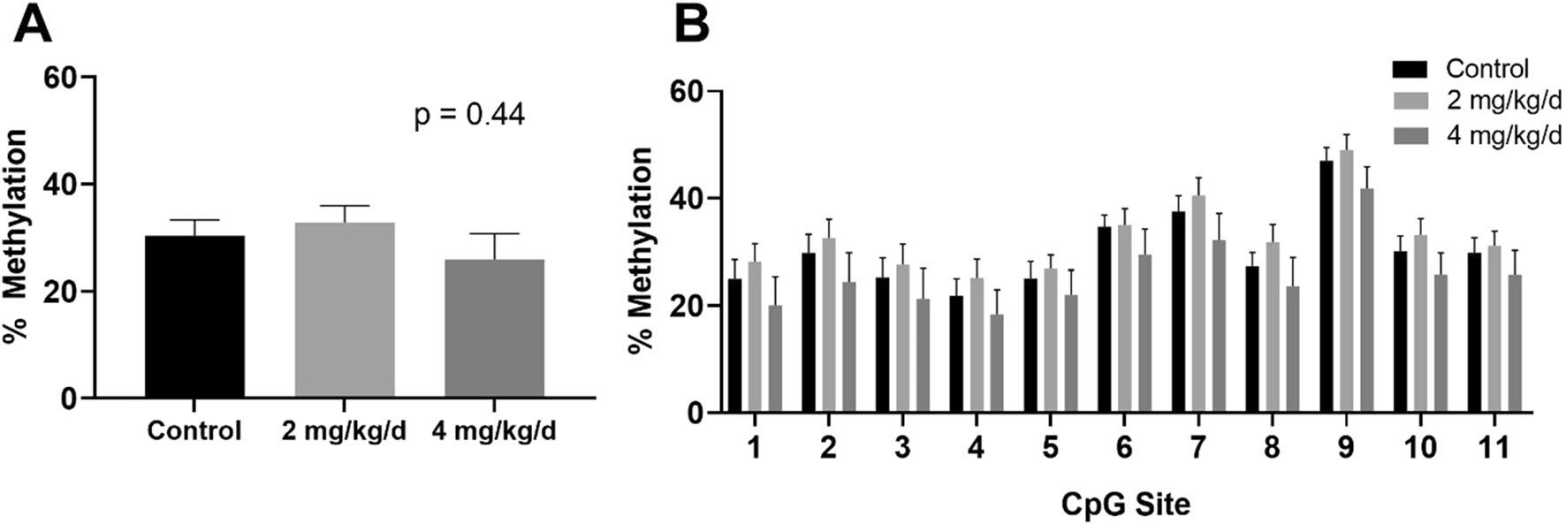
Effect of THC injection (vehicle, THC 2 mg/kg/d, or THC 4 mg/kg/d) on *Por* methylation in sperm. (**A**) Mean and SEM for the control, low dose, and high dose cohorts. ANOVA P value is shown. (**B**) *Por* methylation in sperm in the same population, displayed by individual CpG site. All *p* values were nonsignificant.

Cannabis can be consumed as edibles or inhaled, and we were interested in determining if there were differential effects based on the route of exposure. When adult male rats were exposed to control, 2 mg/kg/d, or 4 mg/kg/d THC via injection, which with more rapid uptake and avoidance of the first-pass liver effect mimics the effects of inhalation, the sperm showed no significant difference in *Por* mean levels of methylation (30.3 vs. 32.8 vs. 25.9% methylation; *p* = 0.44, Fig. 4A). Similarly, there were no significant differences when the sperm were analyzed by individual CpG sites (Fig. 4B). The overall trend for *Por* hypermethylation with THC exposure by oral gavage was indeed observed for the 2 mg/kg/d injected group, but at the higher dose, all CpG sites instead showed hypomethylation.

**Figure 5:**
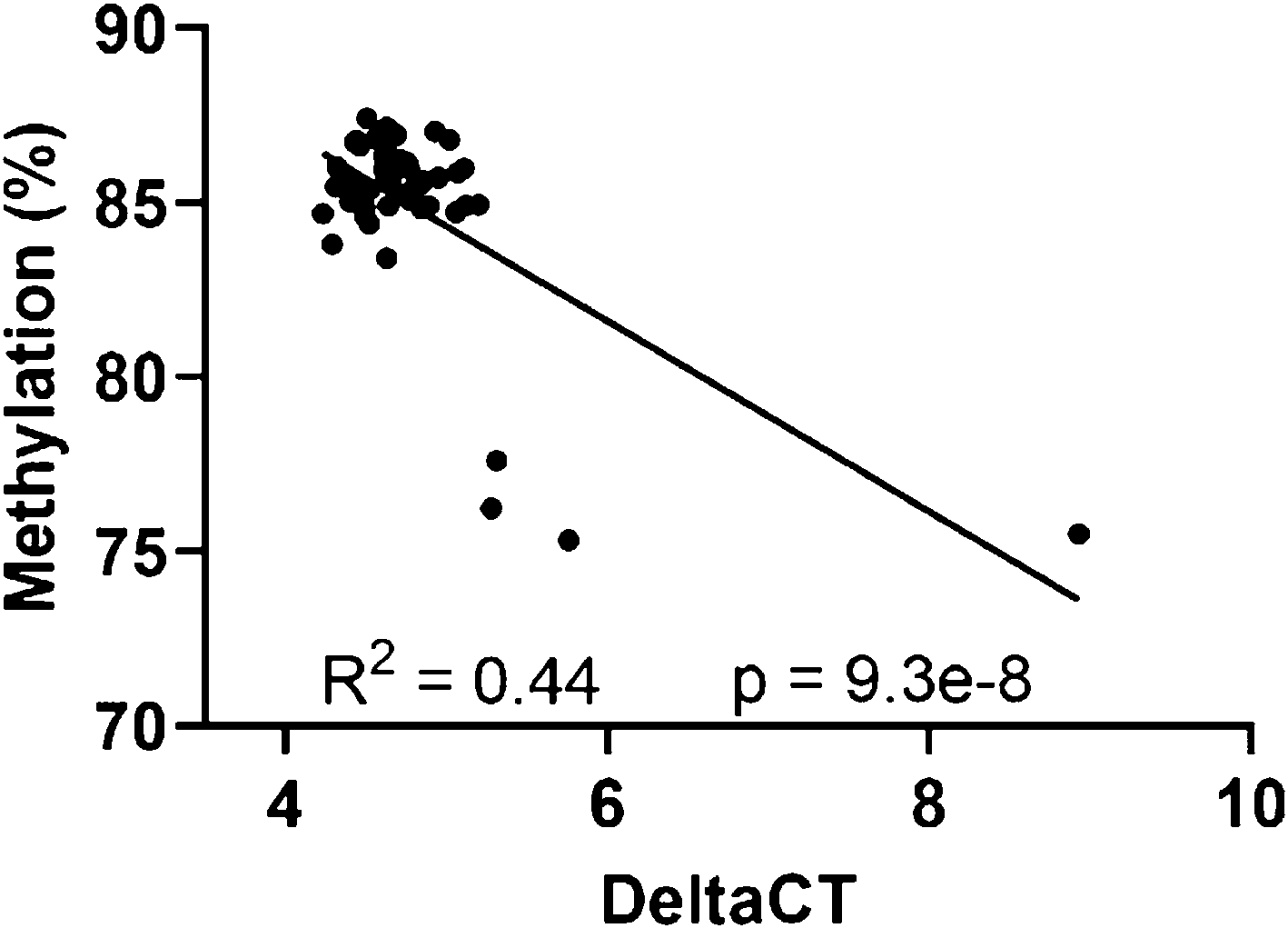
Gene expression-methylation relationship, with deltaCT for brain tissues of the F1 generation shown on the x axis versus *Por* methylation of the same tissues on the y axis.

Since the differential methylation in sperm identified for *Por* following oral gavage is located in an intragenic region of the gene (see Fig. 1), we were interested in determining if there was a relationship between methylation at this region and expression levels of *Por.* Because *Por* is known to be expressed in humans in brain tissues (www.proteinatlas.org) and we were interested in neurobehavioral outcomes in offspring of THC exposed male rats^13^, we evaluated the methylation-expression relationship for *Por* using n = 51 frontoparietal cortex tissues. We found a negative correlation between *Por* methylation at the region of interest and the *Gapdh*-normalized deltaCT values for *Por* (correlation coefficient R = −0.67; *p* < 0.001; Fig. 5), indicating a positive correlation between methylation and gene expression. There were four outliers in the gene expression results, each from different litters, with particularly low *Por* expression. These same four rats also had the lowest methylation at this region of Por (Fig. 3C). Overall, these findings are consistent with what has been shown previously regarding the general positive relationship between intragenic methylation and expression of genes^14,15^.

## Discussion

The results presented herein have shown that there are DNA methylation changes in cytochrome P450 oxidoreductase (*Por*) in the sperm of rats exposed to THC via oral gavage, indicating that ingestion of THC alters epigenetic marks in sperm. *Por* is an 18.6 kb gene located on rat chromosome 12 containing 15 exons. *Por* is highly conserved among vertebrates, and it functions as an electron donor in the Krebs cycle and as an essential component of the steroidogenesis pathway. The Por enzyme impacts the function of more than 50 other enzymes in the cytochrome P450 family^16^ and is critical for the formation of sex steroids, including estradiol, testosterone, cortisol, and aldosterone. Genetic alterations in *Por* can lead to Por deficiency (PORD), which varies in presentation and severity but can cause a range of disorders of sexual development and other developmental disorders, including congenital adrenal hyperplasia, skeletal and cranial abnormalities, facial hypoplasia, and Antley-Bixler syndrome^17,18^. Owing to changes in sex hormone levels, PORD can present in fetal or neonatal life with ambiguous genitalia and later with altered pubertal development and/or infertility. While severe cases of PORD are rare, some researchers postulate that mild or incomplete PORD may be relatively common, such as would be expected with mildly decreased function or depressed expression of the Por enzyme. Previous studies have shown that promoter region methylation of *Por* is associated with decreased gene expression^19^. Ours is the first study that shows that increased methylation at an intragenic region of *Por* located toward the 3′ end of the gene is associated with increased gene expression. There is one CpG island in rat *Por*, located between exons 11 and 12 (chr12: 23,999,545–23,999,785); notably, our identified differentially methylated region (chr12: 23,999,254–23,999,462) lies just outside of this CpG island, located in the “shore” of the island^20^. Interestingly, studies have shown that whereas CpG islands are defined by having the densest concentration of CpG sites, the most vulnerable and consequential CpG sites in terms of cancer and environmental exposures occur in the shore regions^20–24^.

Using robust methods to validate our genome-scale findings, we confirmed that this region of *Por* is hypermethylated in THC-exposed rat sperm after ingestion of THC. We were unable to confirm significant hypermethylation in the other four genes analyzed by targeted pyrosequencing, which we attribute to the inherently high false-discovery rate of using genome-scale methods in which there are millions of comparisons made. We note, however, that the targeted pyrosequencing indeed showed the same directionality of methylation change (hypermethylation with THC exposure) in all genes.

The demonstration of intergenerational heritability of DNA methylation from sperm is challenging in that it requires that the methylation changes do not alter sperm fertilization capability and embryo viability, as well as the methylation change being able to resist the normal process of post-fertilization reprogramming of DNA methylation. In addition, detecting such a change in offspring also requires that the offspring analyzed resulted from fertilization with one of the affected sperm. For a 9% difference in methylation, as we observed for *Por*, and assuming that the differential methylation is occurring in 9% of the sperm population, this means that, all else being equal, there is a 9% chance of a single offspring being derived from that sperm. As stated by Shea et al., “…as fertilization involves the fusion of a single sperm with a single oocyte, modest methylation changes at individual CpGs at best should only alter penetrance of a phenotype across a set of siblings”^25^. To assess for intergenerational inheritance of methylation changes in *Por*, we first analyzed the mean methylation of *Por* in the somatic tissue (brains) of the progeny of THC exposed and unexposed (by gavage) male rats (Fig. 3A). When we analyzed the methylation at each CpG site per individual rat of the F1 generation, we were searching for potential outliers with altered methylation (Fig. 3C), presuming that these individuals may have inherited an epigenetically-altered copy of *Por*. We did find four outliers with particularly low methylation at the first several CpG sites in our region of interest, but two of these outliers were progeny of THC-exposed rats, while two were progeny of control rats. We concluded that there does appear to be an area of hypervariability in *Por* DNA methylation, but we did not find evidence that this was due to parental THC exposure in the animals analyzed. We hypothesize that the inability to detect intergenerational inheritance of these changes could be due to one of three possibilities. First, *Por* may not be a methylation reprogramming “escapee”^10^, and thus its epigenetic marks are erased and rewritten for each subsequent generation; second, sperm with altered methylation in *Por* may be less likely to fertilize oocytes, greatly diminishing the chances of altered methylation being passed to future generations; or third, the sample size of our F1 generation (n = 51) was not sufficiently large to allow for detection of individuals who had inherited an altered epigenetic copy of *Por*, which at best could be estimated to be 9% of the offspring if assuming equal reproductive potential of epigenetically altered vs. unaltered sperm. In this regard, it will be important to determine if the THC-induced methylation changes in sperm across this region of *Por* affect 9% of the total number of sperm (with 91% of the sperm having an unaffected profile in this region within a given male) or if the entirety of the sperm population as a whole exhibits a 9% difference, with every sperm showing a change in methylation for at least one CpG across this region of *Por* (with 0% of the sperm being unaffected). Our present results do not distinguish between these two possible scenarios, but each has implications for the ability to detect intergenerational transmission. To detect the former would require analyzing the specific offspring conceived by one of the affected sperm. Assuming the methylation alteration escapes reprogramming post-fertilization, this would be detectable as a fairly substantial alteration at this region in a small number of the total offspring, likely evident as outliers. The latter scenario would require large numbers of offspring to be able to detect a cumulative difference in methylation based on paternal exposure, since any given pup would show a small alteration at a small fraction of the entire affected region.

Finally, we examined the effect of THC injection, which mimics inhalation, on *Por* methylation in sperm. We did not detect a significant difference between control, low-dose, and high-dose THC exposure (Fig. 4). We postulate that this is likely due to the increased first-pass effect after ingestion of a substance or toxin^11^; with Por being a hepatic enzyme involved in first-pass effect and drug metabolism, it is biologically plausible that *Por* expression would be altered after ingestion rather than inhalation. In support of this hypothesis, our RT-PCR analysis showed a positive correlation between DNA methylation in this region of *Por* and gene expression (Fig. 5).

The strengths of our study include that we performed genome-scale analysis followed by targeted sequencing to confirm epigenetic effects of THC on select genes in rat sperm. We used robust and validated methods which have been shown to be able to detect as small as a 0.5% difference in methylation at a particular CpG site^26^. We examined genes at which multiple CpG sites were altered in the same direction (hypermethylation) to decrease the random epigenetic “noise” that may have been seen by analyzing random single CpG sites throughout the genome. Furthermore, the experimental cohort of rats was treated with a dose of THC chosen to mimic low to moderate human use over a relatively short period of time before conception, rather than using supraphysiologic doses aimed at detecting greater magnitude of effect at the expense of biologically meaningful results. An important limitation of our study, however, was the use of surrogate modes of exposure to THC. As rats are obligatory nose-breathers and smoky air is very stressful, delivery of combusted THC could substantially confound results. Future studies may involve administration of inhaled THC (versus controls with inhaled non-THC smoke) under a light anesthetic in a manner similar to that described by Sarafiana et al.^27^. The oral gavage was performed due to concerns regarding the ability to accurately and equally dose rats exposed via a food source; oral gavage may similarly induce a stress response in rats, but it is important to note that the control rats underwent gavage with vehicle control, so the effect of stress response is assumed to be similar in the two cohorts undergoing gavage. Similarly, the ethanol vehicle used in the gavage cohort of the study may have interacted with gut microbiome of the rats, but we would predict that any differential effects on the gut microbiome would be noted in both the THC and control groups. Of note, recent research has shown that edibles may pose just as high or higher risk to the user than inhaled cannabis^28^, underscoring the importance of examining the safety of THC exposure via various routes, including through gavage to represent ingestion.

In summary, we found hypermethylation in the CpG island shore of cytochrome P450 oxidoreductase (*Por*) in the sperm of rats exposed to THC via ingestion. We report that increased methylation of this region is associated with increased transcription of *Por*, which results in production of an enzyme important in metabolism of drugs and toxins. This effect appears to be present after ingestion of THC but was not found in the arm of our study designed to mimic inhalation, which we hypothesize is due to increased first-pass effect of ingestion of THC through edibles compared with inhalation. And finally, we were unable to detect intergenerational inheritance of the changes in *Por*, possibly due to decreased viability of epigenetically altered sperm, erasure of epigenetic marks in *Por* during methylation reprogramming, or inadequate sample size to detect inheritance of altered sperm, a potentially rare occurrence. Continued research is needed to further elucidate whether these changes can be correlated to the fertility potential of exposed individuals and whether these are potentially heritable by subsequent generations.

## Materials and methods

### Oral gavage (to mimic ingestion) of low-dose THC

**Figure 6:**
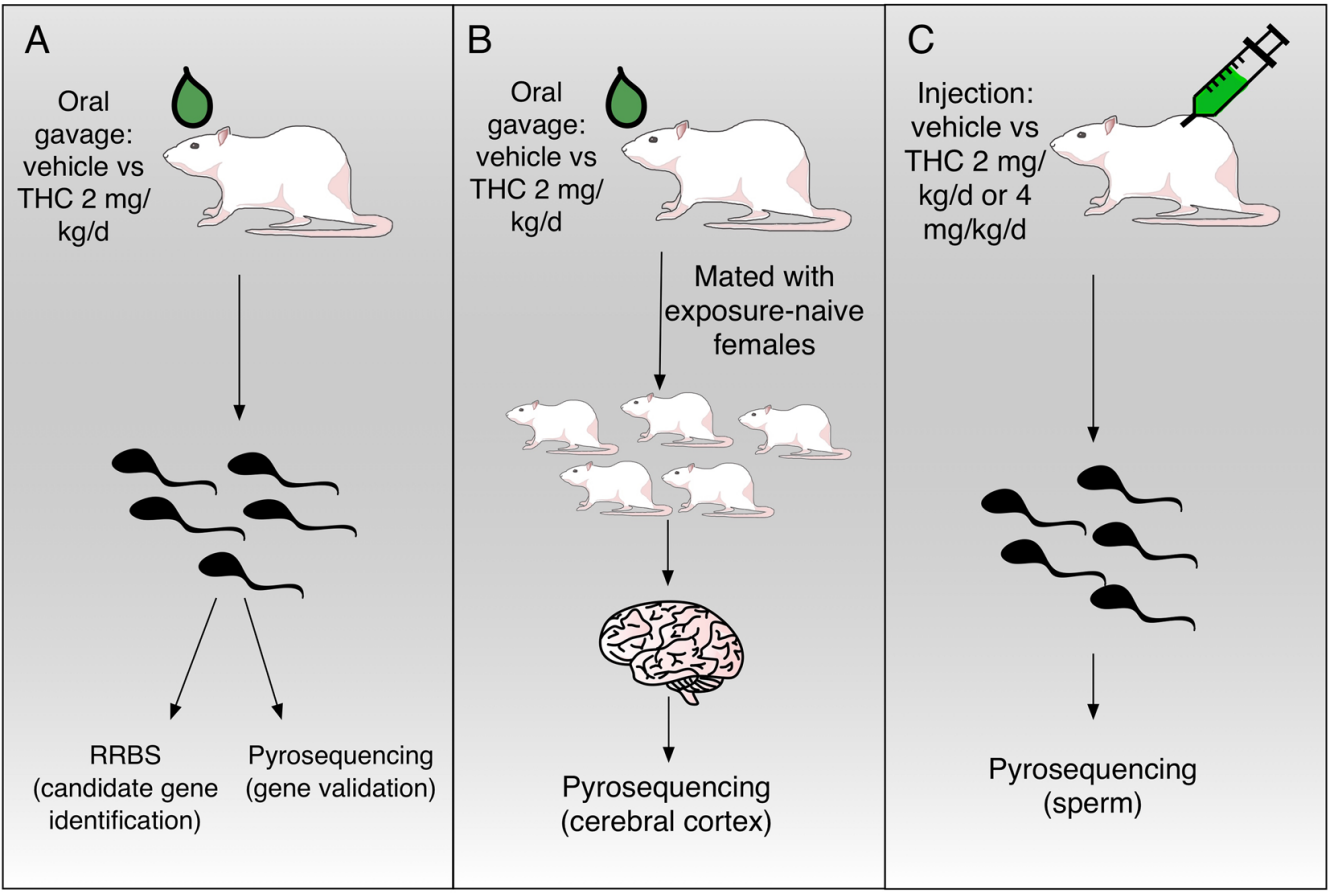
Experimental design. (**A**) Exposure of sperm via gavage: adult male rats were exposed to either vehicle or THC (2 mg/kg/d) via oral gavage. Exposed sperm were isolated, DNA extracted, and reduced representation bisulfite sequencing (RRBS) performed for candidate gene identification. Bisulfite pyrosequencing of the sperm was then carried out to confirm RRBS findings. (**B**) Heritability: Oral gavage-exposed males were mated with exposure-naive females, and pyrosequencing was then performed on the cerebral cortex of the F1 generation. (**C**) Exposure via injection: adult male rats were exposed to either vehicle, THC (2 mg/kg/d), or THC (4 mg/kg/d) via subcutaneous injection. Sperm were then isolated from these exposed males, and bisulfite pyrosequencing was carried out.

This study was reviewed and approved by the Duke Institutional Animal Care and Use Committee and conducted in accordance with federal guidelines. Duke University maintains an animal program that is registered with the USDA, assured through the NIH/PHS, and accredited with AAALAC International. Adult male nine-week-old sexually mature Sprague–Dawley rats were exposed to THC via oral gavage at a dose of 2 mg/kg/d (n = 8) versus vehicle (ethanol) control (n = 7) for 12 days (Fig. 6A). These rats were mated with adult female THC-naïve rats, and their offspring (n = 51) were raised for 24–30 days, sacrificed, and tissues harvested (Fig. 6B). For the original THC-exposed males, epididymal harvest was performed, and the sperm were collected via the swim-out method in PBS to enrich for mature motile sperm. The sperm were stored at −80 °C until used for nucleic acid extraction.

### Subcutaneous injection (to mimic smoke inhalation) of low and high-dose THC

In a separate cohort of rats, sexually mature adult males were exposed to THC via injection (Fig. 6C) at doses of 2 mg/kg/d (n = 4), 4 mg/kg/d (n = 4), or vehicle (4% TWEEN-80 in saline) alone (n = 4). The THC doses were selected to represent light cannabis use, heavy cannabis use, or no use. The mode of injection was chosen to more closely mimic smoke inhalation in terms of the rapid distribution in tissues and onset of effect, as well as the decreased first-pass metabolism and increased bioavailability when compared with oral gavage or ingestion. The exposure time was 28 days. The sperm were collected as described above and analyzed.

### Nucleic acid isolation and bisulfite treatment

DNA was isolated from sperm using the Puregene DNA purification protocol (Qiagen; Valencia CA), in which the sperm sample was subjected to cell lysis and proteinase K digestion followed by treatment with RNAse A solution. Proteins were precipitated and removed, and DNA was isolated and eluted in 30 µl of buffered elution solution. Genomic DNA was quantified using the NanoDrop 2000 (ThermoFisher Scientific; Waltham, MA), and quality was determined using A260/A280 and A260/A230 ratios. If not used immediately, DNA was stored at – 20 °C. Genomic DNA was then treated with sodium bisulfite using the EZ DNA Methylation kit (Zymo Research; Irvine, CA). Bisulfite treatment mutagenizes unmethylated cytosines to uracils, while methylated cytosines are preserved. Following PCR amplification and sequencing, the unmethylated cytosines appear as thymines while methylated cytosines are retained as cytosines in the sequence. The bisulfite-treated DNA was then sent to Zymo Research, where it underwent reduced representation bisulfite sequencing (RRBS). RRBS data underwent QA/QC and analysis as described previously^5^. DNA and RNA were isolated from rat cerebral cortex using the DNA/RNA AllPrep Mini Kit from Qiagen, according to manufacturer’s protocol. RNA was stored at −80 °C until required for use.

### Identification of genes of interest

The RRBS data was used to compare the methylation percentages across CpG sites in THC-exposed rats and controls. Due to limited sample size of the initial study, none of the differences in CpG site methylation between groups passed tests adjusted for multiple comparisons. We therefore used results of student’s t test to identify differentially methylated sites with *p* < 0.05 being considered significant. The list of significant differentially methylated CpG sites was linked to known genes and their upstream promoters and sorted by gene. Genes of interest were selected using the conservative criteria that there were five or more CpG sites showing significant differential methylation and that the magnitude of the difference was greater than 10%. There were five genes meeting this criterion, and these were further analyzed.

### Polymerase chain reaction (PCR) and bisulfite pyrosequencing

Following identification of the differentially methylated genes between users and controls, bisulfite pyrosequencing assays were developed to analyze and validate the methylation status determined by RRBS of the regions of interest for each gene. The UCSC Genome Browser (Rat assembly Jul. 2014 RGSC 6.0/rn6; genome.ucsc.edu^29^) was used to determine the genomic location of each CpG site, and PCR primers were chosen to encompass as many of the identified CpG sites as possible per gene. One primer in each PCR primer set was labeled with a biotin tag. Sequencing primers were chosen to sequence these target regions, with sequence lengths of up to approximately 100 bp. Assay conditions were tested on bisulfite modified rat genomic DNA, and gradient PCR was performed to determine optimal annealing temperature for each primer set. The ability of the pyrosequencing assays to determine methylation status was tested using defined mixtures of commercially available methylated and unmethylated rat DNAs (EpigenDx; Hopkinton, MA). The concentrations of the methylated and unmethylated DNAs were determined using a Qubit (ThermoFisher Scientific), and the methylated and unmethylated DNA were then combined in the necessary ratios to achieve 0%, 25%, 50%, 75% and 100% methylated rat DNA. Bisulfite conversion and pyrosequencing were then carried out in triplicate, and the expected versus measured methylation was plotted using GraphPad Prism Version 8.0 (Graphpad Software; San Diego, CA) (Supplemental Figure S1).

Touchdown PCR was used with three different annealing temperatures for each primer set (see Supplemental Table S2), 5 cycles were performed with each of the first two annealing temperatures, and 55 cycles were performed at the last (lowest) annealing temperature. The PCR products were then confirmed to be single, robust amplicons by electrophoresis on 2% agarose gels run alongside a 50 bp DNA ladder. No-template controls were included for each PCR reaction. The PCR amplicons were then used as the template for bisulfite pyrosequencing of the regions of interest.

Bisulfite pyrosequencing was carried out using a PyroMark MD Q96 pyrosequencer (Qiagen) as previously described^30^. Briefly, the biotin-labeled PCR amplicons were denatured and incubated with streptavidin beads to immobilize the biotin-containing strand on vacuum filtration tips. The complementary (non-biotin-labeled) DNA strands were removed by washing under vacuum. The sequencing primer was then added to the single-stranded DNA, and dNTPs were added sequentially for a sequencing-by-synthesis reaction. The PyroMark software was used to determine the percentage of cytosine versus thymine incorporated at each CpG site relative to the total C + T incorporated, which was used to calculate the methylation percentage for each CpG cytosine. The methylation percentages of the THC-exposed rats were compared to those of the control rats at each CpG site.

### Quantitative real-time reverse transcriptase PCR (RT-PCR)

To determine relative expression levels of *Por*, quantitative real-time reverse transcriptase PCR was carried out using the QuantStudio 6 Flex Real-Time PCR System (Thermo Fischer Scientific). Tissues used included the 51 brains from the rat F1 generation. RNA was extracted from the cerebral cortex of adult F1 rats using the methods described above. A previously validated Taqman probe for *Por* expression (probe ID: Rn00580820_m1; VIC) was multiplexed in a 1:1 ratio with a housekeeping rat gene, *Gapdh* (probe ID: Rn01775763_g1; FAM). The PCR reaction was carried out using a master mix including 4 µl of 50 ng/µl RNA, 1 µl each of the experimental and housekeeping gene probes, 10 µl of qScript 2X one-step qPCR mix, and nuclease-free water for a total volume of 20 µl. Samples were run in duplicate. PCR conditions were 50 °C for 10 m, 95 °C for 1 m, and 40 cycles of 95 °C for 10 s and 60 °C for 1 m. Delta CT values were calculated as the difference between the housekeeping cycle threshold (CT) value and the *Por* CT value.

### Statistical analysis

Analyses were carried out using GraphPad Prism Version 8 (Graphpad). The RRBS results were analyzed for differentially methylated CpG sites using 2-tailed student’s t test, with an alpha value of 0.05 for determination of statistical significance. To confirm the RRBS (genome scale) findings, a one-tailed student’s t test was performed to compare the exposed vs unexposed sperm methylation values obtained by bisulfite pyrosequencing (targeted sequencing), expecting to see similar directionality of methylation changes in the targeted versus genome-scale methods. To elucidate the relationship between methylation of the CpG sites of interest in *Por* and the expression of *Por,* the deltaCT values were plotted against the methylation values. R^2^ and P values were determined using Pearson correlation. For the cohort of rats undergoing injection, three doses were studied (0, 2, or 4 mg/kg/d). Two-way ANOVA was used to test for significant differences between the three groups.

## Supplementary information

Supplementary information

## Data Availability

The RRBS datasets analyzed during the current study are available in the National Center for Biotechnology Information’s Sequence Read Archives database under BioProject Accession PRJNA633085 (https://www.ncbi.nlm.nih.gov/sra/PRJNA633085).
