# Supplementary material for: Epigenetic alterations in cytochrome P450 oxidoreductase (Por) in sperm of rats exposed to tetrahydrocannabinol (THC)
Source: Sci Rep. 2020 Jul 23;10:12251. doi: 10.1038/s41598-020-69204-7 (PMC7378842; doi:10.1038/s41598-020-69204-7)
Supplement: Supplementary file 1 — Supplementary information [file 41598_2020_69204_MOESM1_ESM.pdf]

SUPPLEMENTAL MATERIALS

Epigenetic Alterations in Cytochrome P450 Oxidoreductase (*Por*) in Sperm of Rats Exposed to Tetrahydrocannabinol (THC)

Kelly S. Acharya, MD[1]; Rose Schrott, BS, MS[2]; Carole Grenier, BS[1];

Zhiqing Huang, MD, PhD[1]; Zade Holloway, PhD[3]; Andrew Hawkey. PhD[3];

Edward D. Levin, PhD[2,3]; *Susan K. Murphy, PhD[1].

[1]Department of Obstetrics and Gynecology, Duke University Medical Center, Durham, NC, USA; [2]Duke Nicholas School of the Environment, University Program in Environmental Health, Durham, NC, USA; [3]Department of Psychiatry and Behavioral Sciences, Duke University Medical Center, Durham, NC, USA.

*Corresponding Author:

Susan K Murphy, PhD
Duke University Medical Center
701 West Main Street, Suite 510
Durham, North Carolina 27701
Phone 919.681.3423
susan.murphy@duke.edu

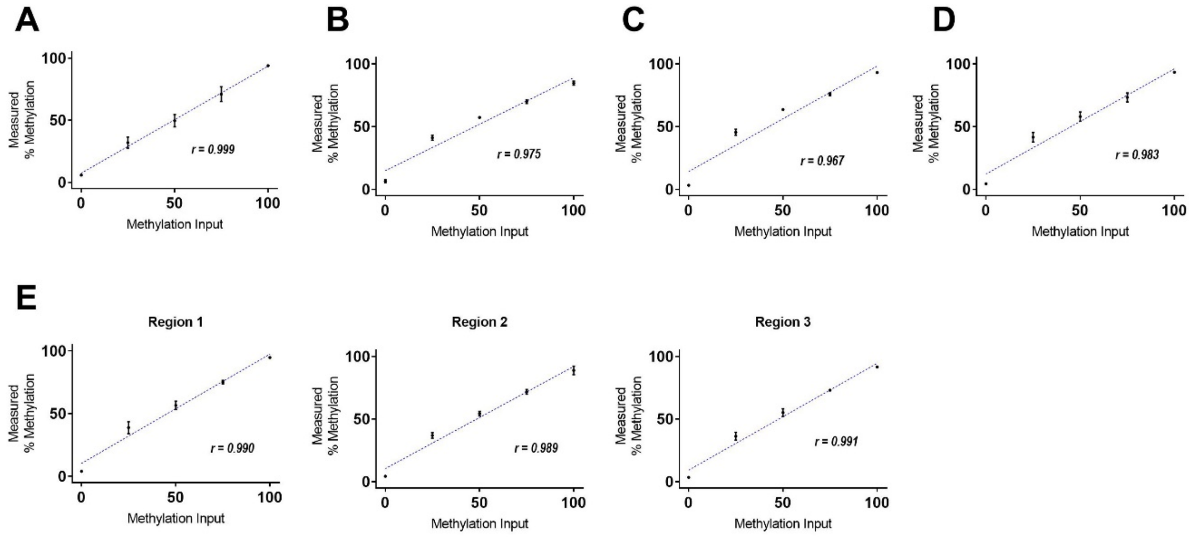

Supplemental Figure 1. Validation curves, showing the mean +/- SD for triplicate measures, for bisulfite pyrosequencing assays of *Adora2a* (A), *Cbx6* (B), *Hipk4* (C), *Mag* (D), and *Por* (three regions) (E). Graphs compare expected DNA methylation on the x axis (based on known methylation percentage of commercially-available unmethylated and methylated rat DNAs) and observed DNA methylation on the Y axis.

Supplemental Table 1: PCR and Pyrosequencing Conditions

Gene	Primer Sequences	PCR Amplicon	PCR Conditions (touchdown annealing temperatures, in ◦C)
Adora2a	F: (Btn)GGTTGATATTGTAGTGGGTGT<br>R: TCCCAAAACCCTACCCTCTATAC<br>S: AATCTAAACCTTATACAC	250 bp	64-61-58
Cbx6	F: GGGAAGTTTTGAGGTTTTATTT<br>R: (Btn)CAAACCAAAATTACCCCCTTATA<br>S: GTTTTTAGTATTAGTGATGT	232 bp	65-62-59
Hipk4	F: AAGGTTTTAGGAATGTAGAGAGT<br>R: (Btn)AATAACTACTTTAAATAAAACCAACATA<br>S: ATTTGAGTTTGTAGGAGGT	132 bp	61-59-56
Mag	F: (Btn)TTGTTTTTATAATTTTTTTGGAAT<br>R: AATAAAACTTAAACCTAACCCC<br>S: CTAAATACACAAACTCCCTC	220 bp	60-58-56
Por	F: GTATTGGGATTGTTTTTTTTATGG<br>R: (Btn)ACCTCACCTTATAAACCTACTCCC<br>S1: TTTTTTTTATGGGTTTTATT<br>S2: GTTTAGGTAAGGAGGTGG<br>S3: GTTAGGATGAGGATTATTTG	293 bp	68-65-62

F = forward primer, R = reverse primer, S = sequencing primer, Btn = Biotin label, bp = base pairs.
